# Internal fixation using fully threaded cannulated compression screws for fresh femoral neck fractures in adults

**DOI:** 10.1186/s13018-022-03005-8

**Published:** 2022-02-20

**Authors:** Kai-xuan Yuan, Fan Yang, Kai Fu, Dao-yu Zhu, Chen-yi Jiang, Dong-xu Jin, Ze-hao Wang, Xiao-yuan Peng, You-Shui Gao, Peng-bo Luo

**Affiliations:** Department of Orthopedics, Shanghai Jiaotong University Affiliated Sixth People’s Hospital, No. 600 Yishan Road, Shanghai, 200233 China

**Keywords:** Femoral neck fracture, Fully threaded screws, Internal fixation, Complications

## Abstract

**Objectives:**

Internal fixation with multiple cannulated compression screws is an optional treatment for femoral neck fracture. Recently, fully threaded cannulated compression screws (FTCCS) have been introduced to fix fresh femoral neck fractures (FNF). The purpose of this study was to investigate the effectiveness of FTCCS.

**Patients and methods:**

Patients with FNF fixed by multiple FTCCS from February 1st, 2014 to August 31st, 2017 were included in this study. They were followed for at least 12 months postoperatively. Nonunion, osteonecrosis of the femoral head (ONFH), fixation failure, reoperation, and femoral neck shortening (FNS) were used to evaluate the outcomes. Risk factors including age, sex, fracture side, fracture displacement, fracture stability, fixation configuration, and screw numbers were analyzed.

**Results:**

A total of 113 patients including 67 males and 46 females with an average age of 48.4 ± 13.4 years were included. The mean duration of follow-up was 27.1 months (range: 12–51 months). The incidence of nonunion, ONFH, fixation failure, and reoperation was 15.9%, 22.1%, 8.8%, and 24.8%, respectively. The rates of nonunion and reoperation were significantly higher in displaced fractures and unstable fractures. And patients with an unstable fracture had a higher risk of internal fixation failure. The median length of FNS was 2.9 mm (interquartile range: 0.9–6.5 mm, range: 0–17.5 mm). Age was a significant risk factor for FNS.

**Conclusions:**

The screw fixation method with FTCCS provided encouraging clinical results which may be a rational choice for the treatment of fresh FNF. Displaced fractures and unstable fractures were attributed to the higher incidence of complications.

*Trial registration*: ChiCTR, ChiCTR1800017200. Registered 17 July 2018-Retrospectively registered, http: www.chictr.org.cn/showprojen.aspx?proj=29182.

## Introduction

Femoral neck fractures (FNF) are a dangerous type of musculoskeletal injury and result in significant morbidity and mortality, accounting for 3.6% of all fractures and 53–56% of hip fractures [1]. The World Health Organization predicts that the incidence of FNF will increase more than threefold by 2050 [2]. Regardless of the degree of displacement, anatomic reduction and stable internal fixation are recommended for patients younger than 70 years with FNF [3, 4] except those who are seriously ill and present excessive surgical risk [5]. Numerous surgical techniques and implants have been developed and used for the treatment of FNF. However, there is no widespread consensus on the optimal fixation techniques and constructs to match the morphology of FNF [6].

For decades, internal fixation using multiple partially threaded cannulated compression screws (PTCCS) remains a standard method for FNF management [7, 8]. These implants hasten to heal through dynamic fracture compression caused by axial loading during weight bearing. However, loss of fixation after closed reduction with three PTCCS is reported to be up to 39% within the first three postoperative months [9], and the subsequent complications including femoral neck shortening (FNS), nonunion, avascular necrosis, and malunion were believed to limit physical function [10].

Biomechanical study demonstrated that the use of screws with increased threads in the metaphysis and the femoral neck might prevent the occurrence of abnormal femoral neck configuration and nonunion of FNF [11]. The construct with a fully threaded positioning screw instead of a partially threaded positioning screw significantly improves the anterior–posterior stiffness and reduces the collapse of the fracture in artificial femurs [12]. Lately, the fully threaded cannulated compression screws (FTCCS) have been introduced to fix large bones in the upper and lower extremities. Compared with traditional PTCCS, the innovative design of the screws includes a patented taper, variable thread pitch, and fully threaded length to provide firm compression and holding power for fractures to minimize hardware loosening and breakage. A very recent study demonstrated that compared with using three traditional PTCCS alone, using fixation of a traditional PTCCS plus two FTCCS improved the clinical outcome [13]. Okcu et al. [14] examined three or four FTCCS with the closed reduction in 22 patients with FNF, and there was no difference of hip scores compared with those using PTCCS.

Nonetheless, to our knowledge, there has been a lack of literature regarding the use of multiple FTCCS alone in the management of FNF with a large sample size and a long follow-up period, to ensure a representative distribution of the population and to be considered representative of groups of patients to whom results will be generalized [14].

The purpose of this study was to investigate the effectiveness of FTCCS in patients with displaced and nondisplaced fresh FNF, mainly analyzing the occurrence of osteonecrosis of the femoral head (ONFH), nonunion, fixation failure, FNS, and reoperation.

## Patients and methods

### Patients

Adult patients admitted to our hospital to treat FNF with FTCCS (7.5 mm, Acumed, Hillsboro, Oregon) between February 1st, 2014 and August 31st, 2017 were included in this study. The time to surgery was 2–4 days after injury. Patients with pathological fractures, associated ipsilateral lower limb fractures, severe spinal or craniocerebral trauma, psychiatric illness, and those younger than 18 years old or treated with other implants were excluded. This study was approved by the ethics committee of our hospital.

**Fig. 1:**
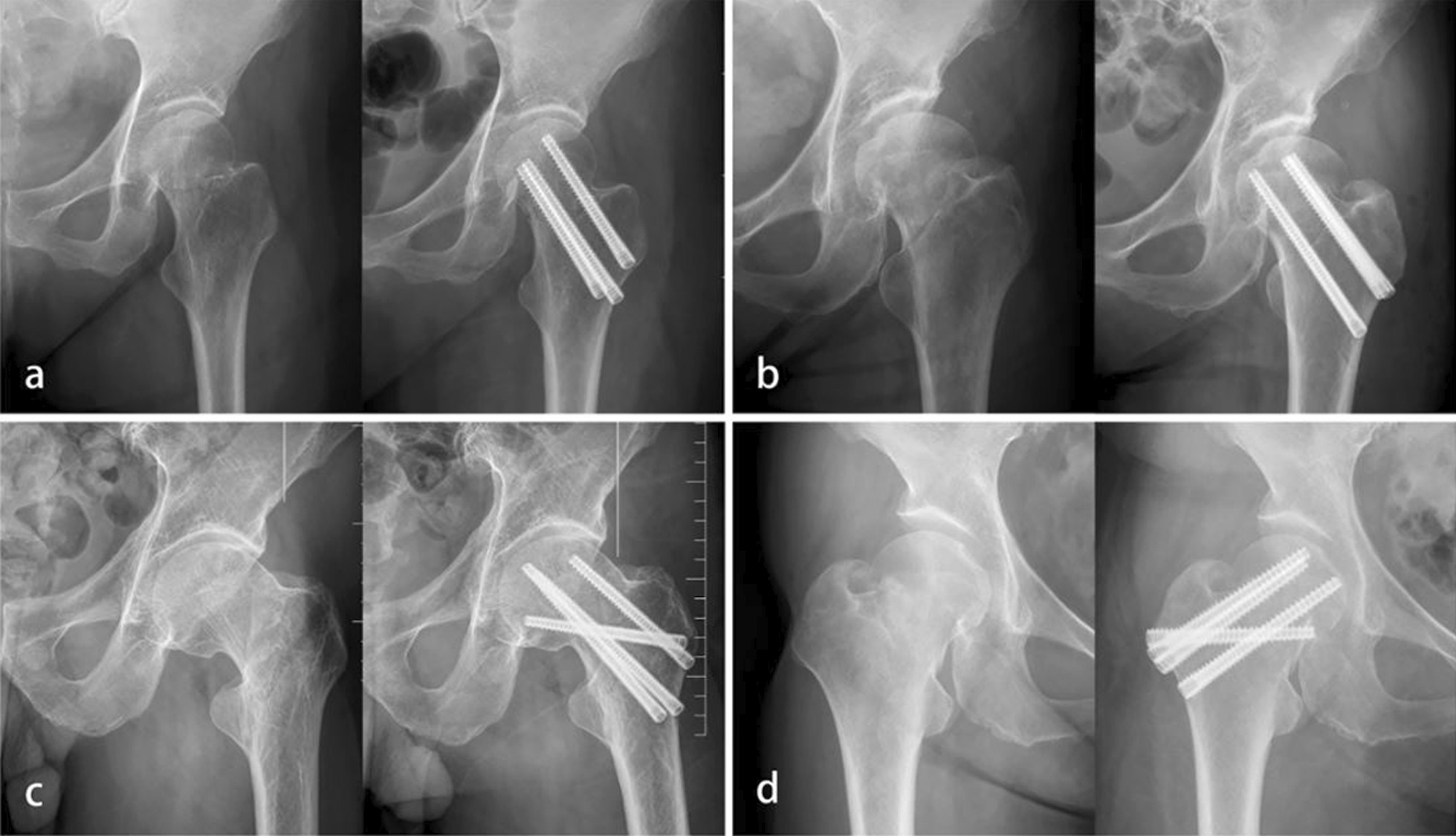
Preoperative and postoperative anterior posterior radiographs of femoral neck fracture treated with fully threaded cannulated compression screws. **a** Fixed by three screws in a triangle configuration. **b** Fixed by three screws in an inverted triangle configuration. **c** Fixed by four screws in a triangle configuration. **d** Fixed by four screws in an inverted triangle configuration

Surgeries were performed by experienced orthopedic surgeons under general anesthesia. Patients received closed reduction in supine position on a fracture traction table. Routine anterior posterior (AP) and lateral planes X-ray were used to evaluate the reduction quality. If the satisfaction of the closed reduction was not gained, open reduction was performed. After successful reduction, the guide wires were inserted in a triangle or in an inverted triangle configuration. Then, the guide wires were measured, screw holes were drilled, and three 7.5 mm FTCCS were inserted. Sometimes one more horizontal screw was used according to the preference of the surgeons (Fig. 1).

Postoperatively, weight bearing was restricted for at least 12 weeks, but movement of the hip and muscle strength exercises on the bed were allowed. Afterward, weight bearing increased gradually.

AP and lateral X-rays of the hip joint were taken at 1.5, 3, 6, and 12 months postoperatively. Patients were also followed up by telephone to investigate their living conditions or reoperations/complications that were not shown in the radiographs.

All patients were followed up with a minimum duration of 12 months. All participants provided informed consent and they could withdraw any time during this study.

### Radiographic analysis

Radiographic data (preoperative and postoperative planes) were collected. Radiographs were used to assess fracture classification, fixation configuration, screw number, fracture healing, and complications including nonunion, ONFH, and internal fixation failure. Fracture classification included two aspects: Pauwels classification [15] and Garden classification [16]. Based on Pauwels classification, fractures were classified as stable (Pauwels type I and II) and unstable (Pauwels type III); according to Garden classification, fractures were distinguished into nondisplaced (Garden type I and II) and displaced (Garden type III and IV).

Nonunion was defined as a clear fracture line existing or displacement of the fracture on the radiographs at 12 months after surgery. ONFH was identified by radiographic signs of Ficat osteonecrosis stage II–IV on any radiograph after surgery [17]. Failure of internal fixation was defined as screws bending, dislocation, or breakage.

**Fig. 2:**
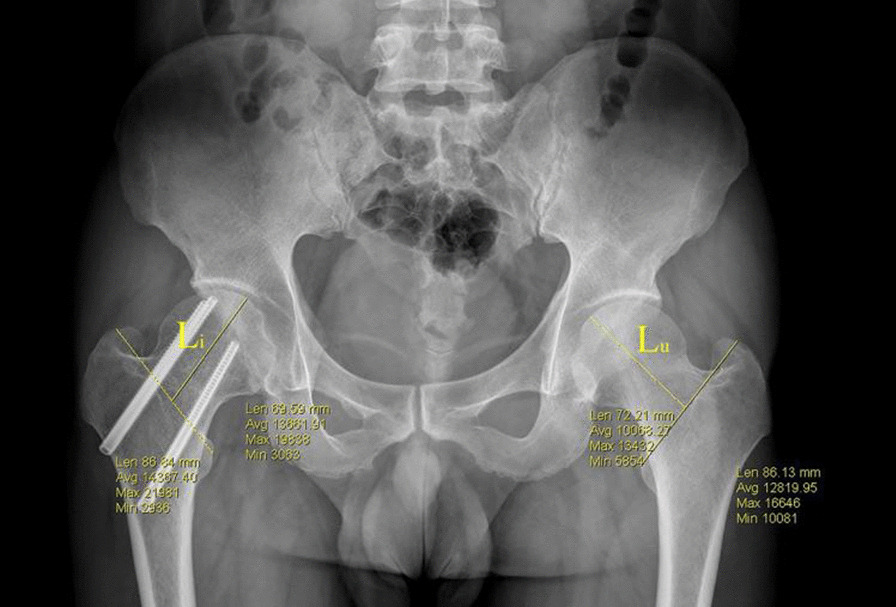
The measurement of femoral neck shortening. On the anteroposterior radiograph, the line was made through the center of the femoral head in the long axis of the femoral neck on both sides. The distance between the tip of the femoral head and the intertrochanteric line was measured. And the length of femoral neck shortening is the distance on the uninjured side minus the distance on the injured side

The FNS was measured by the method described by Yin et al. [18]. In brief, on the anteroposterior radiographs, a line was made through the center of femoral head in the long axis of the femoral neck, and the distance between the tip of the femoral head and the intertrochanteric line was measured on both sides. The length of FNS is the length on the contralateral side minus the length on the fracture side (Fig. 2).

### Statistical analysis

Categorical variables were presented as numbers and percentages (%), and continuous variables were presented as the mean and standard deviation. Differences between groups were compared using the chi-square test or Fisher’s exact test and *t*-test. Statistical analysis was performed using IBM SPSS Statistics (version 24.0; Chicago, USA). A *p* value of ≤ 0.05 (2-sided test) was considered as statistically significant.

## Results

A total of 113 patients treated with FTCCS meeting the inclusion criteria were included in this analysis. There were 67 males and 46 females with an average age of 48.4 ± 13.4 years (range: 19–70 years). Among them, 90 (80%) patients were younger than 60 years old. The mean duration of follow-up was 27.1 ± 10.9 months (range: 12–51 months). In 113 fractures, 68 (60%) were displaced and 44 (39%) were unstable. Sixty-four (57%) fractures were fixed in an inverted triangle configuration and the others were fixed in a triangle configuration. Five (4%) patients were fixed with four screws.

Among the 113 patients, 38 (33.6%) cases of complications were confirmed, including 18 (15.9%) nonunion, 25 (22.1%) ONFH, and 10 (8.8%) fixation failure (two or three of these complications occurred concomitantly in 12 cases). The overall reoperation rate was 24.8% (28 cases), including 12 revision to arthroplasty, four revision to internal fixation, two reinsertion of screws and 10 elective removal of implants. With regard to complication and reoperation incidences, there were no significant differences with respect to age, sex, fracture side, fixation configuration, and screw number (*p* > 0.05). Displaced fractures and unstable fractures were potential risk factors for nonunion and reoperation (Table 1). The rate of nonunion in displaced fractures (24%) was significantly higher than that in nondisplaced fractures (4%) (*p* = 0.007). The nonunion rate in unstable fractures (25%) was higher compared to stable fractures (10%) (*p* = 0.035). The incidence of fixation failure was also significant higher in unstable fractures (18–3%) (*p* = 0.014). And the occurrence of ONFH in displaced fractures was significantly higher than that in nondisplaced fractures (*p* = 0.022).

**Table 1:** Comparison of complication incidence in different groups

	Nonunion (*n* = 18, 15.9%)		ONFH (*n* = 25, 22.1%)		Fixation failure (*n* = 10, 8.8%)		Reoperation (*n* = 28, 24.8%)	
	Present	*P* value	Present	*P* value	Present	*P* value	Present	*P* value
Gender (No, %)								
Male	14 (2)	0.082*	12 (18)	0.193*	8 (12)	0.290*	16 (24)	0.790*
Female	4 (9)		13 (28)		2 (4)		12 (26)	
Fracture side (No, %)								
Left	10 (17)	0.757*	14 (24)	0.667*	6 (10)	0.853*	15 (25)	0.868*
Right	8 (15)		11 (20)		4 (7)		13 (24)	
Fracture displacement (No, %)								
Nondisplaced	2 (4)	0.007*	5 (11)	0.022*	1 (2)	0.093*	5 (11)	0.006*
Displaced	16 (24)		20 (29)		9 (13)		23 (34)	
Fracture stability (No, %)								
Stable	7 (10)	0.035*	13 (19)	0.292*	2 (3)	0.014*	12 (17)	0.023*
Unstable	11 (25)		12 (27)		8 (18)		16 (36)	
Fixation configuration (No, %)								
Triangle	8 (16)	0.920*	12 (25)	0.596*	6 (12)	0.437*	12 (25)	0.950*
Inverted triangle	10 (16)		13 (20)		4 (6)		16 (25)	
Screw number (No, %)								
3	17 (16)	0.587^#^	24 (22)	1.000^#^	10 (9)	1.000^#^	27 (25)	1.000^#^
4	1 (20)		1 (20)		0 (0)		1 (20)	
Age (No, %)								
≤ 60	14 (16)	1.000*	20 (22)	0.960*	7 (8)	0.702*	22 (24)	0.871*
> 60	4 (17)		5 (22)		3 (13)		6 (26)	

The percentages are based on the number with each variable

ONFH: Osteonecrosis of femoral head

^*^P value are based on chi-square test

^#^*P* value are based on Fisher's exact test

For FNS, the median length was 2.9 mm (interquartile range [IQR]: 0.9–6.5 mm, range: 0–17.5 mm). In 113 patients, FNS of > 5 mm occurred in 35 patients, and FNS of > 10 mm occurred in nine patients. FNS of ≥ 5 mm was significantly associated with an older age (48% of patients older than 60 years vs. 27% of the other patients, *p* = 0.050) (Table 2).

**Table 2:** Characteristics of femoral neck shorting

Variable	Total (*n* = 113)	FNS ≤ 5 mm (*n* = 78)	FNS > 5 mm (*n* = 35)	*P* value
Mean age (years)^γ^	48.4 ± 13.4	46.6 ± 14.0	52.5 ± 11.2	0.030
Age (No, %)*				
≤ 60 years	90	66 (73)	24 (27)	0.050
> 60 years	23	12 (52)	11 (48)	
Gender (No, %)*				
Male	67	47 (70)	20 (30)	0.755
Female	46	31 (67)	15 (33)	
Fracture side (No, %)*				
Left	59	37 (63)	22 (37)	0.129
Right	54	41 (76)	13 (24)	
Fracture displacement (No, %)*				
Nondisplaced	45	35 (78)	10 (22)	0.102
Displaced	68	43 (63)	25 (37)	
Fracture stability (No, %)*				
Stable	69	48 (70)	21 (30)	0.877
Unstable	44	30 (68)	14 (32)	
Fixation configuration (No, %)*				
Triangle	49	33 (67)	16 (33)	0.739
Inverted triangle	64	45 (70)	19 (30)	
Screws number (No, %)^#^				
3	108	74 (69)	34 (31)	1.000
4	5	4 (80)	1 (20)	

The percentages are based on the number with each variable

^γ^*P* value was based on *t* test

^*^P value was based on chi-square test

^#^*P* value was based on Fisher's exact test

## Discussion

The purpose of this study was to evaluate the clinical effectiveness of FTCCS in treating adult patients with FNF. The results showed that the overall rate of nonunion, ONFH, and internal fixation failure was 15.9%, 22.1%, and 8.8%, respectively. The nonunion rate of Pauwels type III in our series was 25% and the overall median length of FNS was 2.9 mm. We reckon that FTCCS are reasonable to be used in the treatment of FNF.

PTCCS were commonly indicated for the fixation of displaced and nondisplaced femoral neck in patients with good bone stock [4]. It is well-known that this kind of screw relies on the technique of over drilling the near fragment to provide a gliding hole and then under drilling the far fragment to provide a threaded hole. Interfragmentary compression occurs similarly in cases of the screw and the direction of compression are perpendicular to the axis of the fracture. In addition, sliding will happen as long as the angle is > 20° in relation to its axis [19]. Therefore, one of the advantages is that it enhances fracture healing by compressing the fracture fragments during fixation and eliminating a potential fracture gap by sliding. Clinically, implant sliding can result in FNS and slipping out of the screws, which has been well recognized to be associated with adverse implications and reduced quality of life [12, 20].

To date, there were few studies regarding the optimal length of thread of the screw in the treatment of FNF. Dragoni et al. [11] compared the fixation stability of 16-mm threaded, 32-mm threaded, and fully threaded screws. They demonstrated that the increased number of threads in the metaphysis with these screws might provide additional biomechanical strength to the femoral neck. Downey et al. [21] found that the fully threaded screw biomechanically outperformed the partially threaded screw in shear and proved superior in the initial stiffness and failure strength. According to Schaefer et al.’s results [12], using a fully threaded positioning screw to replace a posterior partially threaded screw could significantly increase bending stiffness and reduce the displacement of the fracture. Parker et al. [22] observed that nonunion rate was decreased in patients with FNF treated with screws with long thread cannulated cancellous screws. These studies indicate that FTCCS might be a valuable choice for FNF fixation.

FTCCS has been introduced to fix large bones in the upper and lower extremities with the advantage of providing strong compression and holding power for fractures to minimize hardware loosening and breakage. In recent years, some alternative ways of FNF fixation using FTCCS have been reported [13, 14, 23]. Weil et al. [23] treated 24 patients with FTCCS and 41 with PTCCS. They revealed that there was no significant difference in nonunion and ONFH rates between the groups. Okcu et al. [14] observed that the use of the same FTCCS as we used in this study increased the nonunion rate (4/22, 18.2%) and the time to union. The nonunion rate of our investigation was comparable to theirs (15.9% vs. 18.2%). Since the configuration of screws was not described in their report, the influence of the configuration of the implants in respect of nonunion could not be evaluated. Meanwhile, they demonstrated that the time to union was significantly longer in patients treated with FTCCS compared with PTCCS, but the hip scores were not significantly different. We did not document these timing data. Logistically, we speculate that a longer time to union may be one disadvantage of this method, but it may not impair the clinical outcomes.

The incidence of nonunion (15.9%), ONFH (22.1%), and reoperation (24.8%) in the current study was similar to that in recent studies using PTCCS (Table 3) [8, 24–32]. Interestingly, the fixation failure rate in our study was significantly lower. Zhang et al. [13] compared the outcomes of 59 patients with vertical FNF treated with three PTCCS alone and a combination of two FTCCS and one PTCCS. They demonstrated that the incidence of fixation failure and nonunion were significantly lower in the combination group. In the current study, fixation failure was detected in 10 cases with an overall rate of 8.8%, and nonunion in 18 cases with a rate of 15.9% (Table 1). Meanwhile, the fixation failure incidence of nondisplaced group was 2% which was lower than that of displaced group (13%), but had no statistically significance based on chi-square test (Table 1). Thus, we believe that fixation with three or four FTCCS is reasonable for the treatment of FNF based on a relatively low incidence of fixation failure.

**Table 3:** The outcomes of FNF fixed by PTCCS in recent studies

Author	Year	Mean follow-up (months)	No. of patients	Nonunion (%)	ONFH (%)	Fixation failure (%)	Reoperation (%)
Liporace F	2008	24	37	18.9	13.5	–	–
Yang JJ	2013	> 12	202	21.8	–	–	–
Thein R	2014	16.8	47	46.8	8.5	–	34.0
Wang SH	2015	36	209	8.4	11.5	–	–
Siavashi B	2015	> 12	28	–	10.7	17.9	28.6
Zhang YL	2016	21.6	46	10.9	17.4	–	–
Chen C	2017	27	44	4.5	9.1	27.3	–
Lu Q	2017	38	41	7.3	9.8	–	22.0
Alshameeri Z	2017	> 12	1279	19.5	5.6	–	19.0
Campenfeldt P	2017	24	170	15.9	14.1	–	25.3
Total				18.3 (374/2044)	7.7 (147/1901)	23.0 (17/74)	20.1 (319/1564)

Classification of FNF has always been done according to the orientation of the fracture line, and relative position of the fracture fragments. The orientation has been described by Pauwels as type I, II, or III [15]. The relative position of fragments has been described by Garden as type I, II, III, and IV [16]. In the present study, nonunion were detected in 4% of the patients with a nondisplaced fracture (Garden type I, II), and in 24% with a displaced fracture (Garden type III, IV). It was reported that the nonunion rate of displaced FNF after internal fixation was 33% in a meta-analysis in which 106 reports were included [33], which is similar to ours. So, our findings indicated that fixation with FTCCS could provide acceptable clinical outcomes in patients with displaced FNF, but more favorable in nondisplaced FNF (Non-union rate, 24% vs. 4%; ONFH rate, 29% vs. 9%; reoperation rate, 34% vs. 11%; Table 1).

It has been well-recognized that FNF with higher shear angles are more unstable, and have a higher tendency of nonunion [8, 33]. Notably, our nonunion rate of Pauwels type III fracture seems to be higher (25%) than that in studies about treating FNF with three PTCCS [8, 26, 28]. The reported nonunion rates were 18.9% [8], 17.4% [26], and 10.9% [28]. Hence, it seems that our method did not provide a superiority compared to the traditional techniques in Pauwels type III FNF. Since FTCCS provides additional strength against shear force and reduces the displacement of the FNF compared with PTCCS, the lack of axially dynamic compression after stable fixation may be the reason [13]. But there was still no biomechanical evidence regarding the compression outcome of FTCCS in FNF. In addition, poor reduction quality may also attribute to the result.

FNS has become a noticeable and common implication after FNF internal fixations, leading to functional impairment [10, 34–36]. Felton et al. [34] had indicated that hip function was significantly poorer when FNS was greater than 10 mm, with a similar result reported by Slobogean et al. [37]. Although some scholars demonstrated that FNS did not impair functional outcome after internal fixation of FNF at mid-term follow-up (5.5 years) [38], it has been widely accepted that increasing FNS was associated with worse hip function. In 2008, Zlowodzki et al. [35] reported that in 56 FNF treated with multiple cannulated screws, FNS greater than 10 mm occurred in 30% of patients, and this shortened group had significantly lower physical function scores. Recent studies showed when FNF were fixed with a sliding hip screw or multiple cancellous screws, the incidence of severe FNS (> 10 mm) was 12–32% [34, 37–40], with a median FNS of 3.1–11 mm [37–39, 41]. In our study, the median length of FNS was 2.9 mm and severe FNS greater than 10 mm was presented only in 8% patients (nine cases), which seemed to decrease FNS compared with traditional fixation methods. These may be caused by the design features of FTCCS. The fully threaded design provided great holding force and made it hard to allow fractures to slide in the axis of the femoral neck. It was further approved by our finding that the fixation failure incidences either in displaced group or nondisplaced was relatively lower than that of using PTCCS(Tables 1, 3). Furthermore, our study found the median length of FNS in patients ≤ 60 years old was significantly shorter than that in patients > 60 years old (2.6 mm vs. 4.9 mm). Meanwhile the incidence of the other complications in the ≤ 60 years old group did not increase. These results demonstrate that FTCCS may be more suitable for relatively young patients. And our previous research has demonstrated that when treating femoral neck nonunion with revision surgery, the success rate was significantly higher when patients had no severe FNS [18]. Consequently, less FNS may offer us the opportunity to achieve better results through revision surgeries, especially for the younger patients.

Some limitations need to be acknowledged in our study. Firstly, our study population was limited to a single center. Thus, we could not generalize to a larger part of China. However, our findings provide evidence for the relative safety and feasibility of the fixation method with FTCCS. Secondly, a control group was lacking, and it is difficult to make a definite value of this method compared to the traditional techniques. Thirdly, the time to surgery of the patientes in our seires was 2–4 days after injury. Thus, the included FNFs were all fresh ones. As we all known, the timing of operation plays a critical role in treatment of FNF. Delayed operation in young patients with FNF may makes it much more difficult to achieve good reduction and fixation because of the aggravated fracture displacement. In addition, the formation of fibrous tissue filling the space between the fracture fragments reduces the possibility to get a satisfied reduction, leads to devastating clinical outcomes. We lack the experiences of treating delayed or old FNF using FTCCS alone. Hence, our results were merely could be adopted in treatment of fresh FNFs. Finally, the bone density was not collected, and accurate measurement may provide different results. Despite these limitations, our findings are clinically significant. The series consisted of a relatively large number of patients and good clinical and radiographic follow-up. All the patients had a mean duration of follow-up of 27 months, and it was adequate to observe development of complications [22].

In conclusion, we found that the screw fixation method with FTCCS provided encouraging clinical results in the treatment of fresh FNF. Unstable fractures and displaced fractures attributed to the higher risk of nonunion, reoperation, and internal fixation failure. These findings demonstrate that FTCCS internal fixation is a proper treatment for FNF.

## Data Availability

Some or all data used during the study are available from the corresponding author by reasonable request.

## Abbreviations

FNF: Femoral neck fractures
FNS: Femoral neck shortening
FTCCS: Fully threaded cannulated compression screws
ONFH: Osteonecrosis of the femoral head
PTCCS: Partially threaded cannulated compression screws
